# Seismic Performance and Cost Analysis of UHPC Tall Buildings in UAE with Ductile Coupled Shear Walls

**DOI:** 10.3390/ma15082888

**Published:** 2022-04-14

**Authors:** Mohammad AlHamaydeh, Mohamed Elkafrawy, Shaziya Banu

**Affiliations:** 1 Department of Civil Engineering, College of Engineering, American University of Sharjah, Sharjah P.O. Box 26666, United Arab Emirates; g00059462@alumni.aus.edu; 2 Ph.D. Program in Material Science and Engineering, College of Arts and Sciences in Collaboration with College of Engineering, American University of Sharjah, Sharjah P.O. Box 26666, United Arab Emirates; b00089515@aus.edu; 3 Structural Engineering Department, Faculty of Engineering, Tanta University, Tanta P.O. Box 31733, Egypt

**Keywords:** UHPC, shear wall, seismic, cost analysis, drift

## Abstract

The superior mechanical characteristics of ultra-high-performance concrete (UHPC) have attracted the interest of many researchers worldwide. Researchers have attempted to perform comparative analyses on the behavior of UHPC versus conventional and high-strength concrete, with their aim being to gain more insights into the difference between different types of concrete. However, the current state-of-the-art revealed no direct comprehensive comparisons between their behaviors in ductile coupled shear walls under seismic loading. This paper explores a comprehensive side-by-side comparison in terms of seismic behavior and cost analysis for four 60-story archetype buildings. The reference building was designed using high-strength concrete with a strength of 60 MPa. The other three archetype variations incorporated three different UHPC grades: 150 MPa, 185 MPa, and 220 MPa. The plan configuration and the lateral force-resisting system (LFRS) were chosen according to the most common practice in the UAE. The main objective is to report the effect of UHPC on the LFRS (ductile coupled shear walls). Moreover, a simplified initial cost analysis (materials and labor) design was performed. The findings of this paper indicate that the use of UHPC is capable of improving the seismic performance behavior of the lateral system as well as reducing the total initial costs.

## 1. Introduction

In recent decades, the number of high-rise buildings has increased all over the world. They are designed and built using innovative structural systems and lighter materials. So, these structures tend to be more flexible with lower damping than previous constructions. However, these buildings are more sensitive to dynamic excitation caused by strong earthquake loads in comparison to older structures. The majority of tall structures are prone to seismic loads and vibrations, and, hence, it is essential to design these structures appropriately. The mass, stiffness, natural period, and damping coefficient are the main dynamic characteristics of these structures. If a structure has a lower natural frequency, then it vibrates significantly under seismic loading. In addition, the structural design response is considered an important factor in the design of tall structures [1]. There has been a significant amount of research on tall buildings with various characteristics such as vertical irregularity [2], irregular plans [3], dual structural systems [4,5], and buildings with a basement [6]. In addition, newly-developed techniques to enhance the seismic performance of RC structures, such as using shape memory alloys with superelastic behavior instead of conventional steel bars, have been investigated in several research studies [7,8,9,10]. From a structural design perspective, this type of building requires concrete with superior properties to withstand the high stresses due to the increasing weight of buildings in addition to the lateral loads effect. Nowadays, conventional and high-strength concrete, which has a compressive strength range from 40 to 70 MPa, is commonly used in the design of these buildings. In general, several research studies have categorized concrete based on its compressive strength into conventional (20 to 50 MPa), high-strength (50 to 80 MPa), high performance (80 to 150 MPa), and ultra-high-performance (150 to 220 MPa) concrete [11,12,13]. Conventional concrete is slowly being replaced with a new, more densified type of concrete called ultra-high-performance concrete (UHPC). This new type of concrete is able to possess more advanced properties in terms of compressive strength, durability, tensile strength, ductility [14,15,16,17,18], the placement of concrete, its brittleness, and the life span of the material. UHPC is considered suitable for bridge decks, piers, strengthening and repair applications, high ductility designs, and elements for blast protection [14,19,20,21,22]. Some other UHPC applications include beam shells [23], column shells [24], and closure joints between prefabricated bridge deck elements [25,26]. Moreover, UHPC has a lower porosity and moisture content compared to other concrete types, which assists in structural rehabilitation [16,18,26,27,28]. However, one of the main limitations of UHPC is that it is perceived as a costly material due to its highly-densified structure. Also, UHPC requires high-temperature curing and fine quartz powder, increasing the associated cost and energy consumption [29]. In addition, Liu et al. reported that UHPC loses most of its strength at high temperatures (higher than 500 °C) [30]. However, fire proofing could be implemented in such cases to improve the fire resistance of UHPC. Thus, this paper compares the seismic performance and analyzes the cost of different models of a 60-story building developed using high-strength concrete and UHPC. In addition, this paper utilizes ductile coupled shear walls to resist the applied seismic loading. Shear walls are usually used as the lateral force resistant system due to their high in-plane stiffness. Almost all shear walls tend to have openings due to the architectural requirements of the building. These openings split the shear walls into two or more slender walls connected using coupling beams. The main advantages of utilizing a coupled shear wall system include the reduction of moment due to the coupling action, the dissipation of seismic energy through the coupling beams over the stories, and a higher lateral stiffness compared to the sum of its individual wall piers [31]. Moreover, hybrid coupled shear walls consisting of reinforced concrete shear walls with steel coupling beams are considered a suitable and appropriate alternative to reinforced concrete shear walls [31,32].

Recently, several studies compared different techniques used to optimize the seismic response of RC structures. In other words, using alternative materials such as FRP bars instead of conventional steel reinforcement led to a significant improvement in different aspects, as reported in [33,34,35,36,37,38,39,40,41,42,43,44,45,46,47,48,49,50,51,52,53,54]. Furthermore, cost-analyses were reported in different seismic applications [55,56,57,58,59,60,61,62,63,64,65,66,67]. They mentioned a significant reduction in costs in addition to the enhancement of the performance of structural elements [68,69,70].

Despite the advanced behavior of UHPC in the construction and design industry, no guidance yet exists for the ways in which a designer can optimize the use of UHPC in structural designs. Numerous studies have investigate UHPC [14,26,71,72,73,74,75,76,77,78,79,80,81,82,83,84,85]. Barnett et al. investigated the effect of the addition of fiber on the strength of UHPC [71,79]. A study conducted by Graybeal, in 2007, proved that UHPC gained over 70 MPa of strength within two days of curing time [72,79]. Another study by Wille et al., in 2011, even produced UHPC with a compressive strength of greater than 210 MPa without steam curing or pressure [73,79]. Khalil et al. studied the effect of using UHP-SHCC in retrofitting some existing structures subjected to repeated loading [74,75,76,77].

Naeimi and Moustafa [78] studied the overall behavior, failing mechanism, and reinforcement values of UHPC, as well as the detailing of its design using finite element modeling and using pushover analysis on DIANA FEA. A bridge with two columns was investigated with the use of UHPC instead of traditional concrete. A parametric analysis was investigated in order to study the effect of different steel ratios and other parameters on the overall behavior of the two piers. To evaluate the difference, a similar model was prepared and analyzed using conventional concrete. The results obtained showed that high-strength steel is essential to utilize the mechanical properties of UHPC to the fullest. The results also showed that the UHPC columns carried more flexure when compared to conventional columns, and this may prove to be economical since smaller cross-sections can be utilized for the columns.

Azmee and Shafiq [80] discussed the importance of using UHPC in the structural field and how it is regarded as the future of concrete. The different design procedures and applications of UHPC are discussed in the paper. UHPC is regarded as the future because it exhibits remarkable properties including very high compressive strength values and self-placing and densifying properties. At the end of the paper, the authors manage to confirm that UHPC possesses better properties than conventional concrete and that it can help to improve the sustainability of buildings. However, it is not very commonly used because of its perceived high cost and challenging design procedure.

Oesch et al. [81] examined the properties and behavior of cementitious material in conventional concrete and UHPC at the micro-scale. This was done in order to better understand the role of these materials and further advance concrete design in the future. The authors identified relationships between design parameters of different materials used in concrete mixing, such as the stiffness parameter and information regarding cracking parameters and the crack volume. A negative gradient relationship was obtained between the stiffness parameters and crack volume for both concrete types.

Valikhani et al. [26] discussed the fact that bridges are always exposed to harsh environmental conditions around them. These harsh conditions can lead to the deterioration of concrete and reduce the service life of the bridge. This paper used UHPC as a repair material to fix issues with bridges in service. The UHPC was used as a sealing layer or crack filler on top of the bridge made of conventional concrete. The issue with this repair strategy is that there is very limited knowledge regarding the bond strength between UHPC and conventional concrete. This paper tested the bond strength using 30 specimens tested under the bi-surface shear test with different surface preparation and roughness degrees. The results concluded that the presence of some roughness between the two surfaces (UHPC and conventional concrete) allows for the growth of a strong bond between the two surfaces involved.

Since the flexural capacity of reinforced columns deteriorates with underground motion due to the crushing of the concrete and the buckling of longitudinal steel bars, research has focused on lessening the damage at the plastic hinge regions of bridge columns in order to develop bridge columns that are seismic-resistant. In their efforts to mitigate this issue, Ichikawa et al. [79] studied the use of locating UHPC segments in plastic hinge regions. Three small-scale columns were tested. Two columns were tested by bilateral cyclic loading and had different plastic hinge detailing. One column had a reinforced concrete core covered in a UHPC jacket. Another column was post-tensioned and had a UHPC hollow-core plastic hinge. Despite the differences in detailing, both columns were designed to have the same strength. The point behind the bilateral cyclic loading was to inflict deformations due to flexure and test the possibility of having torsional modes within the columns. The results proved that the concrete core column successfully carried the applied axial loads to a drift of 6%, while the post-tensioned column carried the applied axial load to a drift of 3.5%. The results showed that the UHPC column proved to have a good performance against seismic loads.

Wille et al. [82] tested the validity of most of the flexural stress and strain models available for calculating the flexure strength of UHPC. The authors considered nine models and tested them by comparing the theoretical results obtained from the models to real experimental data. The reason behind this comparison was the common confusion regarding which model accurately represents the performance of UHPC in flexure. For instance, design guidelines suggest that the way to design ultra-high-performance concrete is by using stress–strain relationships and stress block assumptions. However, assumptions are not enough, and the paper in question demonstrated that ultra-high-performance concrete requires more accurate information. Therefore, the given paper evaluated different combinations of compression and tension stress blocks with experimental data and previous research results in order to find the most precise model for UHPC.

Arora et al. [14] explored the different ways of mixing aggregates and binder to produce sustainable and economical UHPC. Both the aggregates and the binder were selected using the maximum packing density suggested by a compressible packing model to produce UHPC with compressive strengths higher than 150 MPa. In order to achieve UHPC, a cement mass replacement between 30% to 50% was studied. Three aggregate sizes of 6.25 mm, 4.75 mm, and 2.36 mm were selected. The preliminary cost analysis indicated that the use of filler materials and cement replacements reduces material costs. This could be attributed to the reduction in ordinary cement paste content.

Many researchers have studied the performance of UHPC, focusing on its development and behavior from a materials-related perspective. Dong [83] examined the findings obtained from material-related journal papers in a structural design context. To do that, Dong modeled prestressed bridge girders made of UHPC instead of traditional concrete. Dong studied their durability, structural integrity, environmental impact, and cost. After comparing the life-cycle cost of the same bridge modeled using UHPC and normal concrete, the results proved that UHPC is sufficient to prolong the service life of a structure while improving the environmental impact in the long run and reducing the annual cost.

Numerous computational methods exist for describing the behavior of UHPC structures under seismic loading. Wang et al. [84] studied a more straightforward method to model the seismic performance of UHPC bridge columns based on the identified fap-shaped hysteretic model. In this model, the elastic stiffness, yield lateral force, post-yield stiffness ratio, and energy dissipation coefficient are calculated. This method focuses on the yield and ultimate states of the columns. The results of a nonlinear dynamic response state that the identified fap-shaped hysteretic model is suitable for estimating the dynamic responses and maximum drift ratio for UHPC bridge columns under earthquake loading. This simplified method can predict the hysteretic characteristics of UHPC. 

Experimental and numerical investigations were carried out by Ren et al. [85] to describe the performance of UHPC box piers subjected to seismic loading. The failure mode, hysteretic characteristics, energy dissipation, and stiffness degradation were investigated. In order to better understand the performance of UHPC box piers, parametric analysis was analyzed, and the effect of the axial load ratio and the longitudinal reinforcement ratio on the ductility of UHPC box piers was studied. The results showed that the ductility of UHPC box piers decreases with an increase in the longitudinal reinforcement ratio. The test angle plays a significant role in ductility. The ductility is reduced to the minimum when the direction of the lateral loads becomes normal to the diagonal of the cross-section.

This study investigates the feasibility of using UHPC in tall buildings located in moderate seismicity regions in terms of seismic performance and cost-effectiveness. UHPC demonstrates enhanced properties such as a higher compressive strength, a higher ductility and durability, and a better tensile strength [14,15,16,17,18]. Moreover, seismic hazard maps for the United Arab Emirates (UAE) have not been published in international standards yet since it was considered a zero seismic region as per UBC’ 97 code [86]. However, several research studies have performed a seismic hazard assessment of the UAE and reported that most parts of the UAE have moderate to low seismic hazard activities [87]. The coastal areas of the UAE have relatively higher seismic levels as the closest potential earthquake fault is only 100 km away from them [88]. ETABS software was used to model, analyze, and design most of the structural elements, as discussed in more detail in the coming sections. ETABS software has been produced by Computers and Structures, Inc. (CSI), California [89]. Four different buildings were modeled using different compressive strengths of concrete. The first model was for high-strength concrete with a compressive strength of 60 MPa. In comparison, the other three models were for UHPC with compressive strengths of 150, 185, and 220 MPa.

## 2. Research Significance

There is a research gap regarding the influence of UHPC on the seismic performance of tall buildings. In addition, there are no codes or standards developed explicitly for structural design using UHPC. UHPC has been found to be an efficient alternative to conventional concrete. Since the Dubai Municipality published its most recent Seismic Code [90], building officials and structural engineering experts have encouraged engineers to perform seismic vulnerability investigations for the building stock in the UAE. Therefore, this project attempts to evaluate UHPC tall buildings with ductile coupled shear walls as LFRSs in moderate seismicity regions such as the UAE. The cost impact of utilizing UHPC in tall buildings has not been fully explored. Thus, a comparative study of the seismic performance and cost implications of utilizing UHPC in tall buildings will add substantial value to the body of knowledge. To achieve this objective, four archetype 60-story buildings were designed using UHPC with compressive strengths of 60, 150, 185, and 220 MPa. Moreover, an initial cost analysis was also performed to determine if UHPC improves the lateral system seismic performance behavior along with a reduction in the total initial costs. To the best of the authors’ knowledge, there are no studies in the available literature that address this research gap. The findings of this paper may lead to further research on this topic, the usage of UHPC in tall buildings, or ultimately to the development of the design codes or standards for the seismic analysis of tall buildings in the UAE.

## 3. Materials and Methods

Since many high-rise buildings worldwide, especially in the UAE, have a typical number of stories ranging from 40 to 80, the average number of 60 floors was chosen in this research to study the effect of the concrete type. Typically, a 60-story building is considered a tall building, and, particularly in the UAE, plenty of buildings with 60 stories or more exist [91]. However, the authors think that design and ETABS analysis of buildings with greater than 60 stories would be time-consuming and tedious, as numerous iterations were conducted to obtain the most optimum solution for each concrete-type model. Meanwhile, investigating the seismic response of buildings with fewer than 60 stories may not be sufficient to properly understand the seismic effect on tall buildings, especially in the study region. Hence, 60 stories seemed to be the best and most optimum choice for the seismic analysis of tall buildings. In addition, the common concrete grade used in the design of tall buildings is C60 (60 MPa), especially in the UAE. Therefore, the reference model was chosen with a concrete grade of C60. Moreover, UHPC has a compressive strength of 150 to 220 MPa, which can be practically implemented in the construction industry. Therefore, the authors selected three different values, a lowest (150 MPa), a median (185 MPa), and the highest (220 MPa), to be studied in this research.

The purpose of this research paper was to assess and compare difference in terms of structural performance as well as cost when using high-strength concrete or ultra-high-performance concrete in a 60-story building. First, four buildings with different concrete grades (60, 150, 185, and 220 MPa) were modeled and analyzed using ETABS software [89] to meet the seismic serviceability limits (drift-design) according to the ASCE 7-16 standard [92]. For each model, several runs were performed to optimize the structure in terms of the size of elements. Further, design processes for all structural elements were conducted using ETABS [89] and Quick Concrete Wall (QCW) software [93], following IBC-18 [94] and ACI 318-19 [95]. QCW has been produced by Integrated Engineering Software, Inc., Bozeman. In particular, QCW was used in this study, as it provides more detailed design aspects. The comparison between the four models was discussed and summarized in terms of seismic response and cost-effectiveness to provide valuable recommendations at the end of this study.

**Figure 1:**
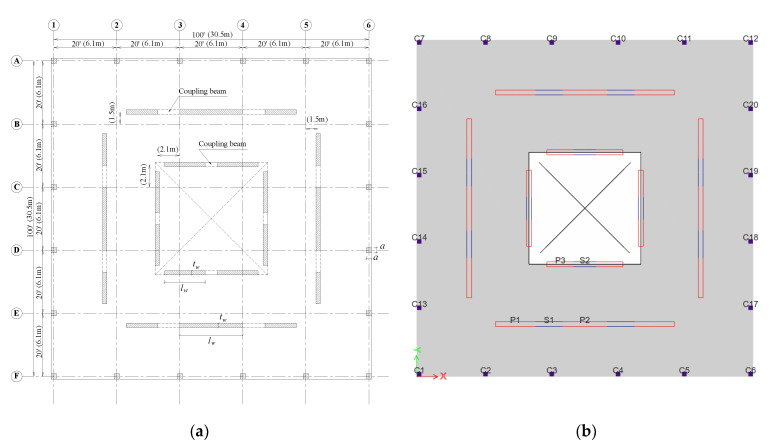
(**a**) Typical floor layout configuration; (**b**) floor plan on ETABS.

According to the formula mentioned in ACI 318-19 [71], section 19.2.2, the concrete elastic modulus was determined and defined in the models considering a concrete unit weight of 2500 kg/m^3^. The high-strength concrete had a compressive strength equal to 60 MPa and the UHPC had three different compressive strength values; 150 MPa; 185 MPa; and 220 MPa. Practically, some engineers consider 60 MPa as a conventional reinforced concrete compressive strength for tall buildings, while others consider it the strength of high-strength concrete. Meanwhile, concrete is considered UHPC if it has a compressive strength greater than or equal to 150 MPa [96]. Other properties for UHPC and HSC were assumed according to Akhnoukh et al. [97,98,99]. Along with the building’s self-weight, it was also subjected to a superimposed dead load of 42.5 psf [2.036 kN/m^2^], a live load of 50 psf [2.394 kN/m^2^], a roof live load of 20 psf [0.958 kN/m^2^] and wall perimeter (cladding) load of 7.5 psf [0.359 kN/m^2^]. Other parameters such as the S_s_ and S_1_ were set equal to 0.935 and 0.365, respectively. These values were obtained based on the S_s_ and S_1_ values for moderate seismic regions. The long period was set equal to 8 s. The building was designed to meet the seismic requirements of ASCE 7-16 and IBC-18. The layout of each floor of the building being modeled is shown in Figure 1a. The 60-story building was modeled on ETABS with a lateral force resisting system (LFRS) of a special concrete shear wall system coupled with ductile coupling beams. The reason for choosing this LFRS is that it is the most common system used in the United Arab Emirates for tall buildings. The gravity system consisted of flat-plate slabs and square columns. Figure 1b shows the typical plan modeled in ETABS and the labels of structural elements that will be discussed in the coming sections. The structural elements in the model (columns, walls, and coupling beams) were designed and analyzed once with the 60 MPa concrete material and once with UHPC using the ETABS and Quick Concrete Wall software programs. The design process of the columns, piers, and spandrels were carried out every five floors for practical purposes. All structural elements were designed using the load combinations shown in Table 1 according to ACI 318-19 [95]. For vertical (flexural) reinforcement and horizontal (shear) reinforcement, structural steel grades of 460 MPa and 420 MPa, respectively, were used.

Gravity loads such as self-weight, dead, live, and roof live loads were evaluated using the equivalent lateral force (ELF) method. A linear static load case type was used for these loads. For the seismic load, four load cases were produced, namely, seismic load (E), seismic drift (E-Drift), response spectrum without eccentricity (E_RS_X), and response spectrum with eccentricity (E_RS_X-ecc). Seismic load (E) and seismic drift (E-Drift) load cases were analyzed using the equivalent lateral force (ELF) method, while response spectrum without eccentricity (E_RS_X) and response spectrum with eccentricity (E_RS_X-ecc) load cases were evaluated for response spectrum analysis. The main difference between the two response spectrum load cases is that the response spectrum without eccentricity (E_RS_X) has no diaphragm eccentricity, but the response spectrum with eccentricity (E_RS_X-ecc) has a diaphragm eccentricity of 0.05.

### 3.1. Gravity System

The design of the gravity system included both a slab and columns design. The thickness of slabs was determined based on the applied gravity loads as well as the span between vertical elements. In other words, a serviceability check (deflection and vibration) and strength design were performed to calculate the minimum slab thickness to minimize the building’s weight. The building’s weight significantly affects the seismic analysis, as will be discussed in the coming sections. Thus, a flat plate with a thickness of 0.2 m was selected for all stories. The building was supposed to move laterally under seismic events. Therefore, the gravity system had to be designed to resist this movement without losing its functionality. ASCE 7-16 [92] mentions the importance of achieving the deformation compatibility between all structural elements in the building. Hence, all columns were designed on an additional moment, which was calculated based on the maximum design drift at every five floors of the building. Stiffness modifiers of 0.7 and 0.25 were applied to the columns and slabs of the models as per ACI 318-19 [95] specifications, respectively.

### 3.2. Lateral Force Resisting System (LFRS)

Special coupled shear walls were used to resist the seismic forces. In ETABS models, all columns were released laterally to consider only the special coupled shear wall in the lateral design. The total dead mass, which includes the self-weight of the entire building and the superimposed loads for finishes, cladding, and brick walls, was used to determine the base shear in ETABS according to ASCE 7-16 [92]. A stiffness modifier of 0.7 was used for the shear walls as per ACI 318-19 [95] specifications. In addition, response spectrum analysis (RSA) was used in the design of the lateral system. The accidental eccentricity of 0.05 was selected according to ASCE 7-16. The scaling factor between the static equivalent force and the response spectrum (RS) force was used to scale the last one up according to ASCE 7-16. Moreover, this standard recommends multiplying the determined scaling factor by (I.g/R), where I is the importance factor, g is the gravity acceleration, and R is the response modification coefficient. Typically, the R value varies between 4.0 and 8.0 [100]. For the moderate seismic region in the UAE, an R-value of 8.0 was considered. According to Ghosh [101], for ductile coupled shear walls, the value of R should be considered as 8.0. For comparison purposes, the value of R was kept constant for both high-strength concrete and UHPC. However, further studies might be conducted to evaluate the validity of this assumption using, for example, nonlinear pushover analysis. Constant seismic parameters were used for high-strength concrete and UHPC models to study the effects of UHPC in detail without the influence of other parameters.

#### 3.2.1. Drift Design

For the drift design, the ETABS software has been used to design the lengths and thicknesses of the shear walls. Several iterations were performed for each model in order to obtain the optimum model in terms of drift-design limits. During these iterations, certain limitations were considered, such as the structure’s natural period being kept in the range of 0.1–0.15 N to prevent the structure from being excessively rigid or flexible. Furthermore, the aspect ratio of the spandrels was kept between 3 to 5. According to Ghosh [101], maintaining an aspect ratio of 3–5 results in flexural dominant behavior and plastic hinging, which is desirable for coupling beams. The cross-sectional aspect ratio of the walls was kept in the range of 6–12 to prevent unnecessary slenderness complications. In addition, the wall lengths were fixed throughout the height of the building, whereas the thicknesses were varied every five floors. The building was designed to ensure the maximum drift in the stories to be less than 2%, according to ASCE 7-16 [92]. The drift values determined from ETABS are elastic and should be converted to the design story drift by multiplying the elastic values by C_d_/I, where C_d_ is the deflection amplification factor taken as per ASCE 7-16 [92].

#### 3.2.2. Strength Design

Both the ETABS and Quick Concrete Wall (QCW) programs were utilized for the strength design. ETABS was used in the design of the columns, shear walls, and coupling beams. Moreover, QCW was used to review the design of shear walls in addition to calculating the boundary elements requirements according to ACI 318-19 [95]. Due to the symmetrical shape of the building, only three piers (piers 1, 2, and 3), two spandrels (spandrels 1 and 2), and three columns (columns 1, 2, and 3) were designed every five floors. The notations of the piers, spandrels, and columns are depicted in Figure 1b.

## 4. Results and Discussion

The subsequent sections report the results of this investigation. Furthermore, they provide a detailed comparison of the seismic performances and cost-effectiveness of the high-strength concrete and the UHPC models.

### 4.1. Seismic Performance

**Figure 2:**
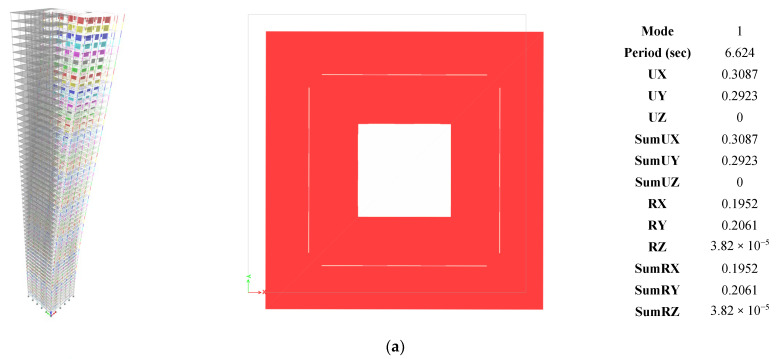

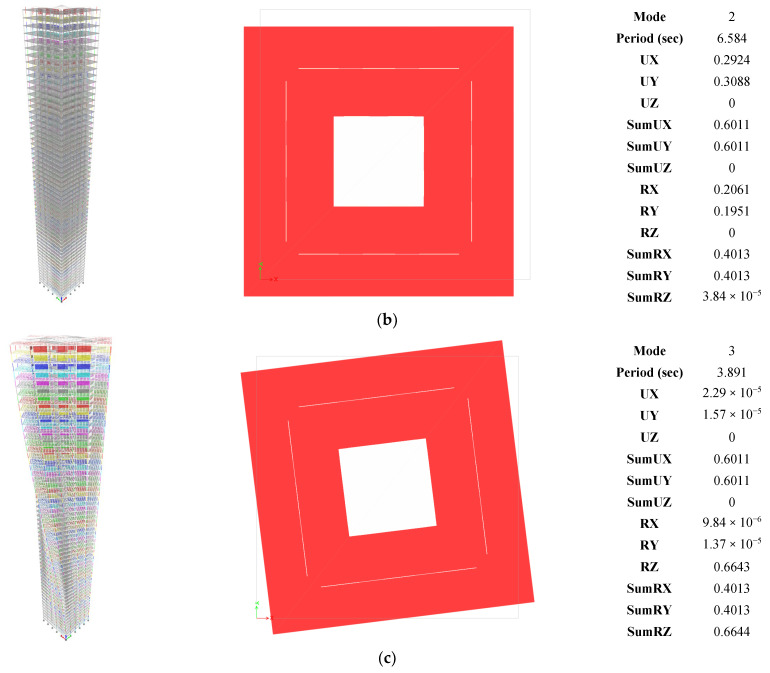
Sample of results for the 60 MPa ETABS model: (**a**) First mode; (**b**) second mode; (**c**) third mode.

Modal analysis was performed using the Eigen method. Figure 2 demonstrates a sample of the first three modes for the high-strength concrete building (60 MPa). The first and second modes were a combination of translations in the x and y directions. The third mode was the torsional mode. The same behavior was observed in all models. Table 2 shows the results of the first 15 modes for the four buildings. ASCE 7-16 [92] recommends using a number of modes that guarantee including at least 90% of the total mass source of the building. The 90% was achieved at mode #15; hence, there was no need for running more modes. However, 30 modes were selected in the ETABS model for each building to increase the accuracy. Table 3 presents the seismic parameters used for the base shear calculation as per ASCE 7-16 [92]. The standard limits the time period used for strength design to a specific limit. On the other hand, the standard allows using the actual time period calculated based on the modal analysis for the drift check. The two values of time periods are tabulated in Table 3. A summary of the shear wall, boundary elements, coupling beams, and columns designs are tabulated in Table 4, Table 5, Table 6 and Table 7, respectively. Table 4 provides the shear wall length (L), thickness (b), vertical reinforcement ratio ($\mu_{V}$), and area of horizontal reinforcement (A_s,H_). It can be noticed that, by increasing the compressive strength of the concrete, most of the cross-sections become smaller. For example, pier P1 at story 1–5 had a length and thickness of 4.55 and 0.65 m, respectively, at 60 MPa concrete strength, but the pier length and thickness reduced to 3.75 and 0.6 m, respectively, at 150 MPa. In addition, the reinforcement ratios also decrease. This reduces the cost of steel reinforcement used in the building and accordingly decreases the amount of work and manpower needed at the construction site. Table 5 summarizes boundary element details such as length (L_be_), thickness (b_be_), and horizontal reinforcement in long (A_s,H,L_) and short (A_s,H,S_) directions. The pier boundary element sizes and the horizontal reinforcement in the long direction were observed to have reduced with an increase in concrete compressive strength. As at story 1–5, the length and thicknesses of pier P1 for 60 MPa, 150 MPa, 185 MPa, and 220 MPa were found to be about 2.05 and 1.3 m, 1.6 and 0.6 m, 1 and 0.6 m, and 0.75 and 0.55 m, respectively. However, the horizontal reinforcement in the short direction increased with an increase in concrete strength, but at higher stories, the requirement of boundary elements reduced with higher concrete strengths. Hence, for the overall building, an increase in concrete strength led to a reduction in the boundary element requirements. Moreover, at a higher concrete strength of 220 MPa, the boundary elements were required only for pier P1 at the base stories, while the P2 and P3 piers did not require any boundary elements throughout the building. It shows that using concrete with a higher compressive strength leads to a significant reduction in the boundary element sizes, which, in turn, enhances the functionality of the free space in the building from an architectural perspective. In general, the reduction in the cross-section of the elements reduces the total building weight, decreasing the foundation’s cost. Table 6 presents the spandrel top (A_s,T_), bottom (A_s,B_), vertical (A_s,V_), and horizontal (A_s,H_) reinforcement areas. In general, the reinforcement areas reduced with an increase in concrete compressive strengths, validating the previous pier design and boundary element design results. A reduction in reinforcement ratios were also noticed with an increase in the story levels. This reduction occurred since higher story levels are susceptible to lower loading compared to base stories. Table 7 provides the column dimensions sizes (length, L and thickness, b) and their vertical reinforcement ratios ($\mu_{V}$) for all stories and compressive strengths. The column sizes and their reinforcement ratios appear to decrease with an increase in concrete strength from 60 MPa to 150 MPa. Therefore, the results of Table 4, Table 5, Table 6 and Table 7 prove that an increase in concrete strength reduces pier, spandrel, and column sizes and their reinforcement requirements.

**Figure 3:**
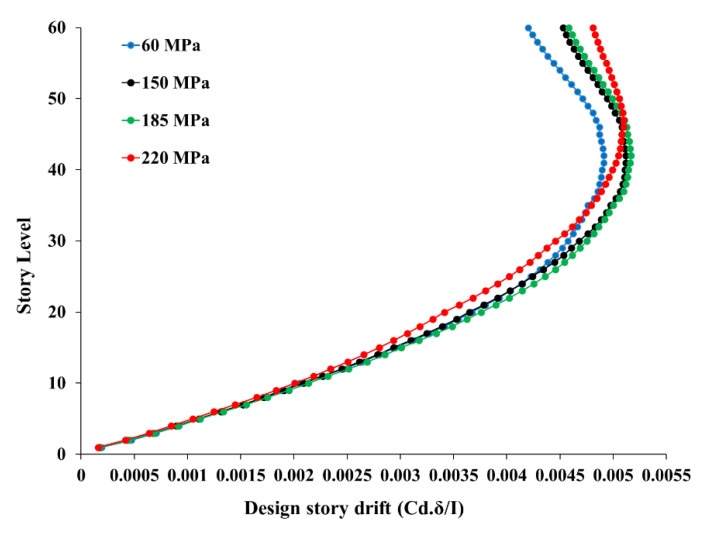
Design story drift.

Figure 3 shows the design story drift at each story for the buildings. The maximum story drift was around 5% near the 40th story. All buildings exhibit almost the same drift behavior, with a significant reduction in cross-sections as well as reinforcement ratios, which leads to better cost-saving. The story drift values were observed to have increased until story 40, and then a slight reduction in drift was observed beyond the 40th story (as shown in Figure 3). The maximum story drifts of 60 MPa, 150 MPa, 185 MPa, and 220 MPa models were found to be about 4.9%, 5.1%, 5.2% and 5.1%, respectively. Therefore, a slight increase of about 4% and 2% was noticed in the maximum story drifts between the 60 MPa to 150 MPa models and the 150 MPa to 185 MPa models, respectively. This proves that UHPC models with compressive strengths of 150 MPa and 185 MPa are more flexible when compared to the high-strength concrete (60 MPa) model. However, a reduction in maximum story drift of about 2% was observed in reaction to the 220 MPa model compared to 185 MPa, which means that the UHPC 220 MPa model is slightly stiffer than the UHPC 185 MPa model. 

**Figure 4:**
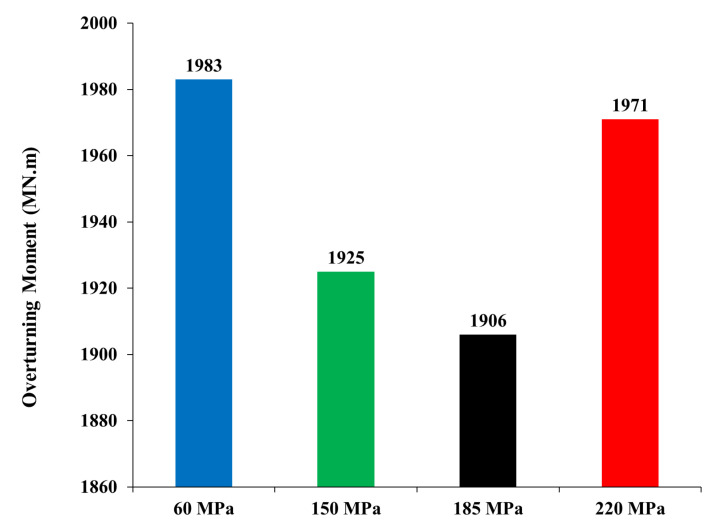
Overturning moment.

**Figure 5:**
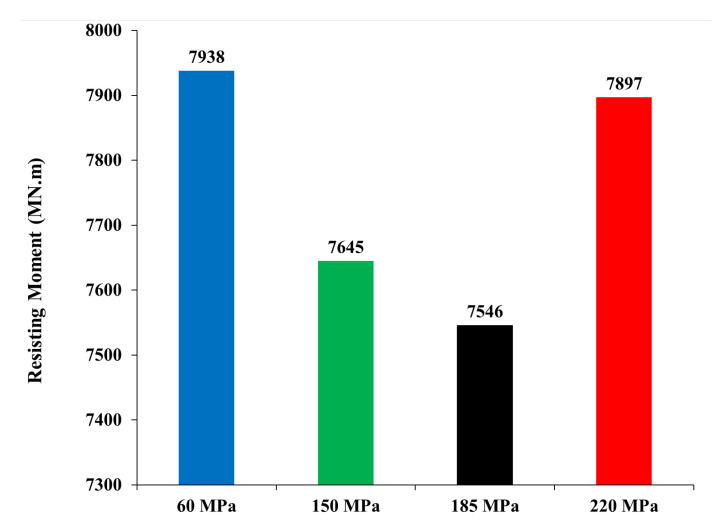
Resisting moment.

**Figure 6:**
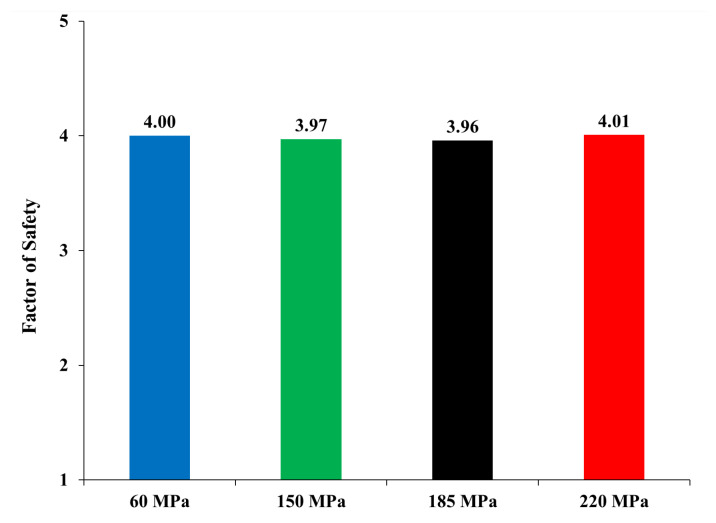
Factor of Safety against overturning $\frac{Resisting\;Moment}{Overturning\;Moment}$.

The induced overturning moment (OTM) was calculated by multiplying each story force, calculated from ETABS, by the corresponding story height to the foundation level. Figure 4 shows the OTM values for the four models. The 150 MPa and 185 MPa models exhibit lower values of OTM. The reduction of the OTM leads to a cost reduction in the foundation design. Moreover, the resisting moment of all four models was calculated and is reported in Figure 5. The resisting moment was determined by multiplying the total weight of each building, which was used in calculating the OTM, by the resisting moment arm. The resisting moment arm was assumed to equal the half-length of the building in addition to 1.0 m, considering that all buildings have a similar plan-foundation, which in most cases is a square raft over piles that has the same dimensions of the building on plan with a cantilever of 1.0 m each side. Furthermore, the weight of the foundation was considered when calculating the resisting moment and was assumed constant for all models, considering the raft having an approximate depth of 3.5 m. A decrease of less than 5.0% was observed in the UHPC models compared to the high-strength concrete model, and this was because the final weight of the UHPC models was slightly (less than 5.0%) lower than the high-strength concrete model. However, the difference in the weights was not significant enough to decrease the resisting moment of the models. The ratio of the resisting moment over the overturning moment is called the factor of safety. Figure 6 presents the factor of safety of the four models, which are greater than 3.5. Therefore, the models are safe against instability due to overturning.

**Figure 7:**
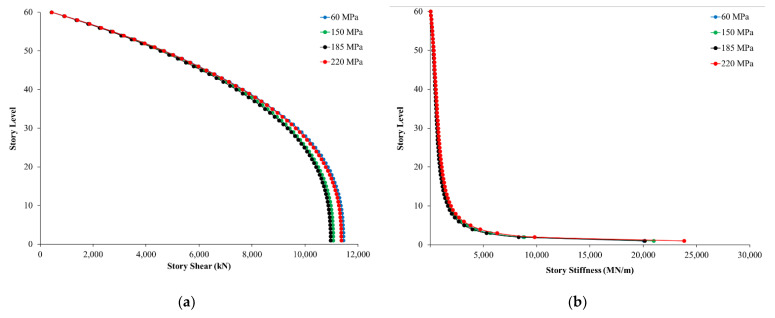
(**a**) Story shear; (**b**) story stiffness.

Figure 7 shows the story shear and story stiffness at each floor of the four buildings. All buildings exhibit very similar values in terms of story shear and stiffness. Both story shear and stiffnesses were reduced with an increase in story levels. Figure 7a clearly shows that at the base story, UHPC 150 MPa, 185 MPa, and 220 MPa have slightly lower shear values in comparison with the high-strength concrete model; this is due to the higher compressive strength of UHPC when compared to high-strength concrete. Story 1 shows cumulative shear values that were observed to have decreased with an increase in concrete strength values (as shown in Figure 7a). Meanwhile, the total stiffness values of story 1 increased with an increase in compressive strengths, except in the case of UHPC 185 MPa, which had a stiffness value nearly the same as the high-strength concrete model (as shown in Figure 7b).

### 4.2. Cost Analysis

In this section, detailed quantity surveying was conducted. The results are summarized in Table 8. The costs are shown in AED in addition to US dollars (USD) in brackets. The cost for concrete was assumed to be 290 AED/m^3^ (78.3 USD/m^3^) and that of UHPC as 550, 600, and 650 AED/m^3^ (148.5, 162, and 175.5 USD/m^3^), for 150, 185, and 220 MPa, respectively, based on the UAE market value at the time of the study. In addition, the cost of steel in the UAE market ranges between 2800 and 3000 AED/m^3^ (756–810 USD/m^3^). Thus, an average value of 2900 AED/m^3^ (783 USD/m^3^) was used in this study. As discussed in El-Tawil et al. [102], UHPC can effectively reduce the dead weight of the structure, as well as indirect costs relating to transportation, manpower, overheads, and formwork costs. The authors of El-Tawil et al.’s paper reported that indirect costs range between 3 and 7 times the direct cost of the reference concrete. The reference concrete refers to the one with the lowest strength value, which in this study is the high-strength concrete. An average value of 5 was considered in this study. Hence, if the cost of the reference concrete is denoted as ‘X’, the additional indirect costs equals 5X. Accordingly, the total cost of each concrete model was equal to 5X in addition to the direct cost obtained for that model. On the other hand, the reduction in indirect costs can be denoted by Z, as proposed by El-Tawil et al. [102]. Moreover, in El-Tawil et al.’s study, the authors recommended values of Z ranging between 10% and 60%, depending on the expected reduction in indirect costs. In this study, reasonable values of Z were proposed and were 40%, 50%, and 60% for the UHPC of 150, 185, and 220 MPa, respectively. These values were chosen considering that higher compressive strengths lead to a higher reductions in indirect costs. The indirect cost of UHPC is calculated as shown in Equation (1):

(1) $$\frac{100-Z}{100}\times indirect\;cost\;of\;conventional\;concrete=\frac{100-Z}{100}\times 5X,$$

**Figure 8:**
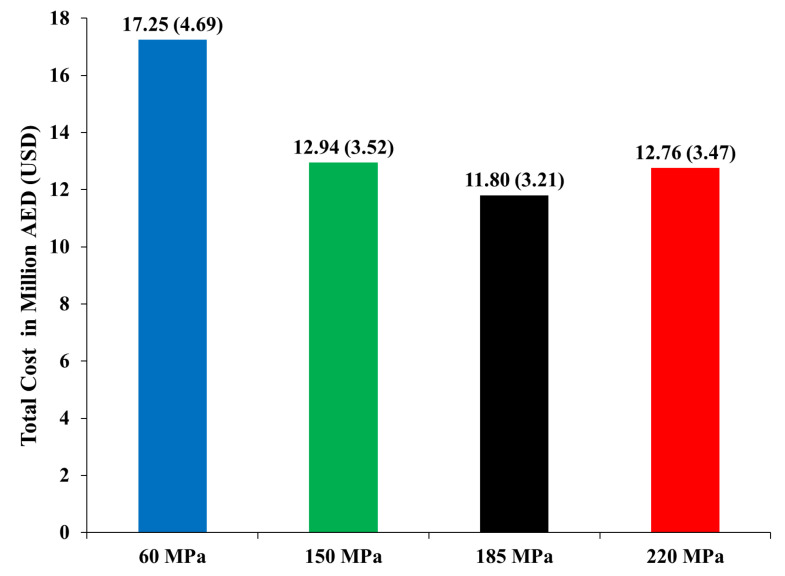
Total cost in AED (USD).

When substituting the cost of the reference concrete (HSC) in place of X, the indirect costs were evaluated as AED (USD) 11,430,022 (3,111,818), 5,868,620 (1,597,729), 4,708,592 (1,281,912), and 4,273,390 (1,163,428) for the high-strength concrete and UHPC 150, 185, 220 MPa, respectively. The results show that using UHPC can save up to around AED (USD) 5,500,000 (1,497,413) from the indirect cost, as shown in Table 8. In addition, Figure 8 shows the total cost of each building. The total cost of UHPC was determined to be lower than the high-strength concrete model, as shown in Table 8. It is worth mentioning that initial construction and curing costs were also considered in the indirect costs, so even with the inclusion of all indirect costs, the UHPC models were found to be relatively less expensive. In general, increasing the strength of the concrete results in more compact cross sections in reinforced concrete structural members. This produces a smaller volume of concrete. Table 8 also shows that the concrete volume follows this trend except for when increasing from 185 to 220 MPa, which results in a higher concrete volume in comparison to 185 MPa. This is attributed to the larger dimensions of P3 in this model compared to other models. This is a clear indication that when using the 220 UHPC, the drift limit states govern the strength limit states (the shear wall design is controlled by the drift rather than the strength). As such, the shear walls sizes are increased to satisfy the drift limitations and thus cause a greater volume of concrete when compared to 185 MPa.

This proves that the model used for UHPC 220 MPa may not be the most optimum design and further modeling could be performed in order to achieve a more efficient and optimum design for UHPC 220 MPa. However, since the total cost of the UHPC 220 MPa model is lower than the high-strength concrete model and the UHPC 150 MPa model, the UHPC 220 MPa design is in fact not overly inefficient and can be used for the analysis of this study. A significant reduction in total cost in AED and USD can be noticed clearly in Figure 8. A reduction in the total cost of 25%, 31%, and 26% can be achieved by using UHPC of 150, 185, and 220 MPa, respectively, rather than high-strength concrete.

## 5. Conclusions

In this paper, four models for a 60-story building were developed to quantify the effect of using UHPC as a replacement for high-strength concrete. The modal analysis results and the design summary of the columns and the special concrete coupled shear walls were presented and discussed. The main findings of this study are summarized as follows:

- All buildings exhibited a similar seismic response but with smaller structural elements in UHPC buildings.
- A reduction in the dead weight of the building was observed, in addition to a lower value for overturning moments, for the UHPC building compared to the high-strength concrete building, which results to cost-saving in the design of the foundation.
- The results showed that by increasing the strength of concrete from high-strength concrete of 60 MPa to the UHPC of 150, 185, and 220 MPa, the total initial costs were reduced by 25%, 31%, and 26%, respectively.
- In terms of seismic performance and cost analysis, the best results were observed when using the UHPC of 185 MPa. Therefore, this study recommends using the UHPC in tall buildings instead of high-strength concrete.
- The reduction in the cost can be attributed to the decrease in the dead load of the structure, the smaller sizes of the required structural elements for lateral load resistance (ductile coupled shear walls), and lower manpower transportation costs.
- Future nonlinear analysis (e.g., pushover) is highly recommended to validate the assumed parameters (such as the response modification coefficient; R) for UHPC tall buildings.

## Figures and Tables

**Table 1:** Load combinations.

Name	Formula	Description
U1	1.4D	Dead
U2	1.2D + 1.6L	Dead + Live
U3	1.2D + 1.6L + 0.5Lr	Dead + Live + Roof Live
U4	1.2D + 1.0L + 1.6Lr	Dead + Live + Roof Live
U5	1.3D + 0.5L ± 1.0E	Dead (max) + Live ± Seismic
U6	0.8D ± 1.0E	Dead (min) ± Seismic

**Table 2:** Modal analysis results.

Mode	Period (s)				Direction	SumUX	SumUY	SumUZ
	60 MPa	150 MPa	185 MPa	220 MPa				
1	6.624	6.813	7.007	6.56	UX + UY	0.3087	0.2923	3.82 × 10^−5^
2	6.584	6.794	6.995	6.556	UX + UY	0.6011	0.6011	3.84 × 10^−5^
3	3.891	4.798	5.144	2.862	RZ	0.6011	0.6011	0.6644
4	1.704	1.691	1.694	1.502	UX + UY	0.6887	0.6869	0.6644
5	1.697	1.688	1.693	1.502	UX + UY	0.7746	0.7746	0.6644
6	1.209	1.355	1.389	0.957	RZ	0.7746	0.7746	0.7914
7	0.786	0.746	0.745	0.642	UX + UY	0.8105	0.8102	0.7915
8	0.783	0.745	0.745	0.642	UX + UY	0.846	0.846	0.7915
9	0.628	0.64	0.641	0.511	RZ	0.846	0.846	0.8538
10	0.473	0.434	0.431	0.368	UX + UY	0.8665	0.8663	0.8538
11	0.472	0.433	0.431	0.368	UX + UY	0.8867	0.8867	0.8538
12	0.395	0.379	0.375	0.322	RZ	0.8867	0.8867	0.8909
13	0.327	0.291	0.288	0.248	UX + UY	0.8999	0.8997	0.8909
14	0.326	0.291	0.288	0.248	UX + UY	0.9129	0.9129	0.8909
15	0.279	0.257	0.252	0.228	RZ	0.9129	0.9129	0.9157

**Table 3:** Seismic parameters for base shear calculation.

	Design Criteria	C_t_ and X	S_S_	S_1_	T_L_ (s)	F_a_	F_v_	S_DS_	S_D1_	R	Ω	C_d_	I	Period Used (s)	Coeff Used	Weight Used (kN)	Base Shear (kN)
60 MPa	Strength	0.02; 0.75	0.935	0.365	8	0.8	0.8	0.5	0.2	8	2.5	8	1	4.457	0.021	482,812	10,593.5
	Drift													6.584	0.003	482,812	1784.4
150 MPa	Strength													4.457	0.021	464,769	10,197.6
	Drift													6.794	0.003	464,769	1664.6
185 MPa	Strength													4.457	0.021	458,677	10,063.9
	Drift													6.995	0.003	458,677	1595.5
220 MPa	Strength													4.457	0.021	480,253	10,537.4
	Drift													6.556	0.003	480,253	1782.4

**Table 4:** Shear walls design summary.

Floors	Pier Label	60 Mpa				150 Mpa				185 Mpa				220 Mpa			
		L (m)	b (m)	***ρ_V_* (%)**	A_s,H_ (mm^2^/m)	L (m)	b (m)	***ρ_V_* (%)**	A_s,H_ (mm^2^/m)	L (m)	b (m)	***ρ_V_* (%)**	A_s,H_ (mm^2^/m)	L (m)	b (m)	***ρ_V_* (%)**	A_s,H_ (mm^2^/m)
1–5	P1	4.55	0.65	0.84	1630	3.75	0.6	2.11	1500	3.65	0.6	2.58	1500	3.7	0.55	1.8	1380
	P2	3.7	0.65	0.25	1630	4.1	0.6	0.25	1500	4.05	0.6	0.25	1500	3.3	0.55	0.25	1380
	P3	2.9	0.5	0.25	1250	2.7	0.45	0.25	1130	2.45	0.4	0.25	1000	3.8	0.5	0.98	1250
6–10	P1	4.55	0.6	0.46	1500	3.75	0.55	1.61	1380	3.65	0.55	2.05	1380	3.7	0.5	1.36	1250
	P2	3.7	0.6	0.25	1500	4.1	0.55	0.25	1380	4.05	0.55	0.25	1380	3.3	0.5	0.25	1250
	P3	2.9	0.45	0.25	1130	2.7	0.4	0.25	1000	2.45	0.35	0.25	880	3.8	0.45	0.93	1130
11–15	P1	4.55	0.5	0.25	1250	3.75	0.5	1.16	1250	3.65	0.5	1.55	1250	3.7	0.5	0.91	1250
	P2	3.7	0.5	0.25	1250	4.1	0.5	0.25	1250	4.05	0.5	0.25	1250	3.3	0.5	0.25	1250
	P3	2.9	0.35	0.25	880	2.7	0.35	0.25	880	2.45	0.3	0.25	750	3.8	0.45	0.7	1130
16–20	P1	4.55	0.45	0.25	1130	3.75	0.45	0.72	1130	3.65	0.45	1.06	1130	3.7	0.5	0.52	1250
	P2	3.7	0.45	0.25	1130	4.1	0.45	0.25	1130	4.05	0.45	0.25	1130	3.3	0.5	0.25	1250
	P3	2.9	0.3	0.25	750	2.7	0.3	0.25	750	2.45	0.25	0.25	630	3.8	0.45	0.44	1130
21–25	P1	4.55	0.4	0.25	1000	3.75	0.4	0.25	1000	3.65	0.4	0.5	1000	3.7	0.4	0.37	1000
	P2	3.7	0.4	0.25	1000	4.1	0.4	0.25	1000	4.05	0.4	0.25	1000	3.3	0.4	0.25	1000
	P3	2.9	0.3	0.25	750	2.7	0.25	0.25	630	2.45	0.25	0.25	630	3.8	0.35	0.25	880
26–30	P1	4.55	0.4	0.25	1000	3.75	0.35	0.25	880	3.65	0.4	0.25	1000	3.7	0.4	0.25	1000
	P2	3.7	0.4	0.25	1000	4.1	0.35	0.25	880	4.05	0.4	0.25	1000	3.3	0.4	0.25	1000
	P3	2.9	0.3	0.25	750	2.7	0.25	0.25	630	2.45	0.25	0.25	630	3.8	0.35	0.25	880
31–35	P1	4.55	0.4	0.25	1000	3.75	0.3	0.25	750	3.65	0.35	0.25	880	3.7	0.35	0.25	880
	P2	3.7	0.4	0.25	1000	4.1	0.3	0.25	750	4.05	0.35	0.25	880	3.3	0.35	0.25	880
	P3	2.9	0.3	0.25	750	2.7	0.25	0.25	630	2.45	0.25	0.25	630	3.8	0.3	0.25	750
36–40	P1	4.55	0.3	0.25	750	3.75	0.25	0.25	630	3.65	0.3	0.25	750	3.7	0.3	0.25	750
	P2	3.7	0.3	0.25	750	4.1	0.25	0.25	630	4.05	0.3	0.25	750	3.3	0.3	0.25	750
	P3	2.9	0.25	0.25	630	2.7	0.25	0.25	630	2.45	0.25	0.25	630	3.8	0.3	0.25	750
41–45	P1	4.55	0.25	0.25	630	3.75	0.25	0.25	630	3.65	0.25	0.25	630	3.7	0.25	0.25	630
	P2	3.7	0.25	0.25	630	4.1	0.25	0.25	630	4.05	0.25	0.25	630	3.3	0.25	0.25	630
	P3	2.9	0.25	0.25	630	2.7	0.25	0.25	630	2.45	0.25	0.25	630	3.8	0.25	0.25	630
46–50	P1	4.55	0.2	0.25	500	3.75	0.2	0.25	500	3.65	0.2	0.25	500	3.7	0.2	0.25	500
	P2	3.7	0.2	0.25	500	4.1	0.2	0.25	500	4.05	0.2	0.25	500	3.3	0.2	0.25	500
	P3	2.9	0.2	0.25	500	2.7	0.2	0.25	500	2.45	0.2	0.25	500	3.8	0.2	0.25	500
51–55	P1	4.55	0.2	0.25	500	3.75	0.2	0.42	500	3.65	0.2	0.4	500	3.7	0.2	0.5	500
	P2	3.7	0.2	0.25	500	4.1	0.2	0.25	500	4.05	0.2	0.25	500	3.3	0.2	0.41	500
	P3	2.9	0.2	0.25	500	2.7	0.2	0.25	500	2.45	0.2	0.25	500	3.8	0.2	0.25	500
56–60	P1	4.55	0.2	0.76	500	3.75	0.2	1.02	500	3.65	0.2	0.97	500	3.7	0.2	1.29	500
	P2	3.7	0.2	0.47	500	4.1	0.2	0.64	500	4.05	0.2	0.62	500	3.3	0.2	1.23	500
	P3	2.9	0.2	0.25	500	2.7	0.2	0.48	500	2.45	0.2	0.76	500	3.8	0.2	0.45	500

Table notations: L (wall length), b (wall thickness), *ρ_V_* (vertical reinforcement ratio), A_s,H_ (horizontal reinforcement).

**Table 5:** Boundary element design summary.

Floors	Pier Label	60 Mpa				150 Mpa				185 Mpa				220 Mpa			
		L_be_ (m)	b_be_ (m)	A_s,H,L_ (mm^2^/m)	A_s,H,S_ (mm^2^/m)	L_be_ (m)	b_be_ (m)	A_s,H,L_ (mm^2^/m)	A_s,H,S_ (mm^2^/m)	L_be_ (m)	b_be_ (m)	A_s,H,L_ (mm^2^/m)	A_s,H,S_ (mm^2^/m)	L_be_ (m)	b_be_ (m)	A_s,H,L_ (mm^2^/m)	A_s,H,S_ (mm^2^/m)
1–5	P1	2.05	1.3	47,502	7137	1.6	0.6	45,708	16,408	1	0.6	34,752	20,272	0.75	0.55	30,530	21,930
	P2	1.4	1.3	32,292	7137	0.55	0.6	14,943	16,408	NA	NA	NA	NA	NA	NA	NA	NA
	P3	1.13	1	25,857	5382	0.6	0.45	16,408	12,013	0.45	0.4	14,842	13,032	NA	NA	NA	NA
6–10	P1	2.05	1.2	47,502	6552	1.3	0.55	36,918	14,943	0.9	0.55	31,132	18,462	0.7	0.5	28,380	19,780
	P2	1.4	1.2	32,292	6552	0.55	0.55	14,943	14,943	NA	NA	NA	NA	NA	NA	NA	NA
	P3	1.13	0.9	25,857	4797	0.6	0.4	16,408	10,548	0.5	0.35	16,652	11,222	NA	NA	NA	NA
11–15	P1	2.05	1	47,502	5382	1.05	0.5	29,593	13,478	0.8	0.5	27,512	16,652	NA	NA	NA	NA
	P2	1.53	1	35,217	5382	NA	NA	NA	NA	NA	NA	NA	NA	NA	NA	NA	NA
	P3	1.3	0.7	29,952	3627	0.6	0.35	16,408	9083	0.5	0.3	16,652	9412	NA	NA	NA	NA
16–20	P1	1.95	0.9	45,162	4797	0.9	0.45	25,198	12,013	0.75	0.45	25,702	14,842	NA	NA	NA	NA
	P2	1.5	0.9	34,632	4797	NA	NA	NA	NA	NA	NA	NA	NA	NA	NA	NA	NA
	P3	1.33	0.6	30,537	3042	0.65	0.3	17,873	7618	0.55	0.25	18,870	7770	NA	NA	NA	NA
21–25	P1	1.8	0.8	41,652	4212	1.85	0.4	53,033	10,548	NA	NA	NA	NA	NA	NA	NA	NA
	P2	1.48	0.8	34,047	4212	NA	NA	NA	NA	NA	NA	NA	NA	NA	NA	NA	NA
	P3	1.13	0.6	25,857	3042	0.65	0.25	17,873	6153	NA	NA	NA	NA	NA	NA	NA	NA
26–30	P1	1.94	0.6	33,462	4212	0.7	0.35	19,338	9083	NA	NA	NA	NA	NA	NA	NA	NA
	P2	1.67	0.6	28,782	4212	NA	NA	NA	NA	NA	NA	NA	NA	NA	NA	NA	NA
	P3	1.24	0.45	21,177	3042	0.55	0.25	14,943	6153	NA	NA	NA	NA	NA	NA	NA	NA
31–35	P1	1.6	0.6	27,612	4212	NA	NA	NA	NA	NA	NA	NA	NA	NA	NA	NA	NA
	P2	1.37	0.6	23,517	4212	NA	NA	NA	NA	NA	NA	NA	NA	NA	NA	NA	NA
	P3	1	0.45	17,082	3042	NA	NA	NA	NA	NA	NA	NA	NA	NA	NA	NA	NA
36–40	P1	1.7	0.45	29,367	3042	NA	NA	NA	NA	NA	NA	NA	NA	NA	NA	NA	NA
	P2	1.47	0.45	25,272	3042	NA	NA	NA	NA	NA	NA	NA	NA	NA	NA	NA	NA
	P3	0.9	0.45	15,327	3042	NA	NA	NA	NA	NA	NA	NA	NA	NA	NA	NA	NA
41–45	P1	1.57	0.45	27,027	3042	NA	NA	NA	NA	NA	NA	NA	NA	NA	NA	NA	NA
	P2	1.34	0.45	22,932	3042	NA	NA	NA	NA	NA	NA	NA	NA	NA	NA	NA	NA
	P3	0.47	0.375	7722	2457	NA	NA	NA	NA	NA	NA	NA	NA	NA	NA	NA	NA
46–50	P1	1.24	0.45	21,901	3146	NA	NA	NA	NA	NA	NA	NA	NA	NA	NA	NA	NA
	P2	1.17	0.45	21,204	3224	NA	NA	NA	NA	NA	NA	NA	NA	NA	NA	NA	NA
	P3	0.6	0.2	7616	2176	NA	NA	NA	NA	NA	NA	NA	NA	NA	NA	NA	NA
51–55	P1	0.8	0.2	9424	1984	NA	NA	NA	NA	NA	NA	NA	NA	NA	NA	NA	NA
	P2	0.55	0.2	6936	2176	NA	NA	NA	NA	NA	NA	NA	NA	NA	NA	NA	NA
	P3	NA	NA	NA	NA	NA	NA	NA	NA	NA	NA	NA	NA	NA	NA	NA	NA
56–60	P1	NA	NA	NA	NA	NA	NA	NA	NA	NA	NA	NA	NA	NA	NA	NA	NA
	P2	NA	NA	NA	NA	NA	NA	NA	NA	NA	NA	NA	NA	NA	NA	NA	NA
	P3	NA	NA	NA	NA	NA	NA	NA	NA	NA	NA	NA	NA	NA	NA	NA	NA

Table notations: L_be_ (boundary element length), b_be_ (boundary element thickness), A_s,H,L_ (horizontal reinforcement in long direction), A_s,H,S_ (horizontal reinforcement in short direction).

**Table 6:** Spandrels design summary.

Floors	Spandrel Label	60 MPa				150 MPa				185 MPa				220 MPa			
		A_s,T_ (mm^2^)	A_s,B_ (mm^2^)	A_s,V_ (mm^2^/m)	A_s,H_ (mm^2^/m)	A_s,T_ (mm^2^)	A_s,B_ (mm^2^)	A_s,V_ (mm^2^/m)	A_s,H_ (mm^2^/m)	A_s,T_ (mm^2^)	A_s,B_ (mm^2^)	A_s,V_ (mm^2^/m)	A_s,H_ (mm^2^/m)	A_s,T_ (mm^2^)	A_s,B_ (mm^2^)	A_s,V_ (mm^2^/m)	A_s,H_ (mm^2^/m)
1–5	S1	7110	6259	5250	1630	5702	5169	4590	1500	6270	5832	5780	1500	5021	4541	4680	1380
	S2	2244	1818	1370	1250	2333	2015	1460	1130	2109	1856	1490	1000	4688	4268	5070	1250
6–10	S1	6902	6353	5560	1500	5456	4987	4560	1380	6058	5643	5720	1380	4629	4266	4470	1250
	S2	1944	1563	1160	1130	2041	1791	1330	1000	1802	1624	1310	880	4573	4171	5080	1130
11–15	S1	6063	5492	4910	1250	5084	4634	4320	1250	5703	5270	5440	1250	4399	4045	4210	1250
	S2	1956	1586	1480	880	2044	1695	1540	880	1766	1441	1460	750	4506	4122	5020	1130
16–20	S1	5854	5275	4800	1130	4772	4316	4130	1130	5405	4964	5230	1130	4003	3652	3700	1250
	S2	1721	1373	1320	750	1941	1601	1560	750	1628	1309	1410	630	4909	4476	5570	1130
21–25	S1	5483	4899	4560	1000	4513	4058	4000	1000	5040	4602	4940	1000	4106	3730	4090	1000
	S2	1562	1254	1150	750	1765	1451	1490	630	1542	1270	1360	630	3703	3387	4250	880
26–30	S1	5088	4557	4250	1000	4173	3727	3770	880	4679	4237	4520	1000	3956	3544	3960	1000
	S2	1464	1179	1050	750	1583	1306	1290	630	1514	1256	1340	630	3581	3281	4100	880
31–35	S1	4573	4098	3800	1000	3720	3282	3410	750	4352	3892	4210	880	3366	3025	3400	880
	S2	1266	1012	820	750	1548	1293	1280	630	1343	1160	1120	630	3174	2900	3690	750
36–40	S1	3823	3390	3310	750	3181	2761	2960	630	3418	2991	3320	750	2969	2648	3030	750
	S2	1144	915	820	630	1528	1299	1290	630	1399	1198	1270	630	2849	2602	3260	750
41–45	S1	3224	2829	2850	630	2730	2325	2470	630	2842	2445	2780	630	2444	2148	2500	630
	S2	1020	819	690	630	1299	1119	1010	630	1299	1160	1150	630	2315	2095	2660	630
46–50	S1	2504	2157	2260	500	2045	1679	1850	500	2177	1811	2140	500	1920	1657	1980	500
	S2	756	581	500	500	993	895	770	500	996	928	880	500	1696	1503	1920	500
51–55	S1	1726	1441	1500	500	1404	1074	1140	500	1457	1135	1280	500	1389	1164	1300	500
	S2	529	358	500	500	847	648	500	500	890	713	500	500	1025	1012	960	500
56–60	S1	674	589	500	500	1005	618	500	500	1061	856	660	500	865	618	500	500
	S2	529	500	500	500	433	296	500	500	408	263	500	500	866	688	500	500

Table notations: A_s,T_ (top reinforcement area), A_s,B_ (bottom reinforcement area), A_s,V_ (vertical reinforcement area), and A_s,H_ (horizontal reinforcement area).

**Table 7:** Columns design summary.

Floors	Pier Label	60 MPa		150 MPa		185 MPa		220 MPa	
		Cross Section (m)	$\rho_{V}(\%)$	Cross Section (m)	$\rho_{V}(\%)$	Cross Section (m)	$\rho_{V}(\%)$	Cross Section (m)	$\rho_{V}(\%)$
1–5	C1	0.55 × 0.55	1.45	0.50 × 0.50	1	0.50 × 0.50	1	0.50 × 0.50	1
	C2	0.55 × 0.55	4.04	0.50 × 0.50	1	0.50 × 0.50	1	0.50 × 0.50	1
	C3	0.55 × 0.55	2.95	0.50 × 0.50	1	0.50 × 0.50	1	0.50 × 0.50	1
6–10	C1	0.55 × 0.55	1	0.50 × 0.50	1	0.50 × 0.50	1	0.50 × 0.50	1
	C2	0.55 × 0.55	2.49	0.50 × 0.50	1	0.50 × 0.50	1	0.50 × 0.50	1
	C3	0.55 × 0.55	1.77	0.50 × 0.50	1	0.50 × 0.50	1	0.50 × 0.50	1
11–15	C1	0.50 × 0.50	1.69	0.45 × 0.45	1	0.45 × 0.45	1	0.50 × 0.50	1
	C2	0.50 × 0.50	3.72	0.45 × 0.45	1	0.45 × 0.45	1	0.50 × 0.50	1
	C3	0.50 × 0.50	3.29	0.45 × 0.45	1	0.45 × 0.45	1	0.50 × 0.50	1
16–20	C1	0.50 × 0.50	1	0.45 × 0.45	1	0.45 × 0.45	1	0.40 × 0.40	1
	C2	0.50 × 0.50	1.68	0.45 × 0.45	1	0.45 × 0.45	1	0.40 × 0.40	1
	C3	0.50 × 0.50	1.51	0.45 × 0.45	1	0.45 × 0.45	1	0.40 × 0.40	1
21–25	C1	0.50 × 0.50	1	0.45 × 0.45	1	0.40 × 0.40	1	0.40 × 0.40	1
	C2	0.50 × 0.50	1	0.45 × 0.45	1	0.40 × 0.40	1	0.40 × 0.40	1
	C3	0.50 × 0.50	1	0.45 × 0.45	1	0.40 × 0.40	1	0.40 × 0.40	1
26–30	C1	0.45 × 0.45	1	0.40 × 0.40	1	0.40 × 0.40	1	0.40 × 0.40	1
	C2	0.45 × 0.45	1	0.40 × 0.40	1	0.40 × 0.40	1	0.40 × 0.40	1
	C3	0.45 × 0.45	1	0.40 × 0.40	1	0.40 × 0.40	1	0.40 × 0.40	1
31–35	C1	0.45 × 0.45	1	0.40 × 0.40	1	0.40 × 0.40	1	0.40 × 0.40	1
	C2	0.45 × 0.45	1	0.40 × 0.40	1	0.40 × 0.40	1	0.40 × 0.40	1
	C3	0.45 × 0.45	1	0.40 × 0.40	1	0.40 × 0.40	1	0.40 × 0.40	1
36–40	C1	0.45 × 0.45	1	0.40 × 0.40	1	0.40 × 0.40	1	0.40 × 0.40	1
	C2	0.45 × 0.45	1	0.40 × 0.40	1	0.40 × 0.40	1	0.40 × 0.40	1
	C3	0.45 × 0.45	1	0.40 × 0.40	1	0.40 × 0.40	1	0.40 × 0.40	1
41–45	C1	0.45 × 0.45	1	0.40 × 0.40	1	0.40 × 0.40	1	0.40 × 0.40	1
	C2	0.45 × 0.45	1	0.40 × 0.40	1	0.40 × 0.40	1	0.40 × 0.40	1
	C3	0.45 × 0.45	1	0.40 × 0.40	1	0.40 × 0.40	1	0.40 × 0.40	1
46-50	C1	0.45 × 0.45	1	0.40 × 0.40	1	0.40 × 0.40	1	0.40 × 0.40	1
	C2	0.45 × 0.45	1	0.40 × 0.40	1	0.40 × 0.40	1	0.40 × 0.40	1
	C3	0.45 × 0.45	1	0.40 × 0.40	1	0.40 × 0.40	1	0.40 × 0.40	1
51–55	C1	0.45 × 0.45	1	0.40 × 0.40	1	0.40 × 0.40	1	0.40 × 0.40	1
	C2	0.45 × 0.45	1	0.40 × 0.40	1	0.40 × 0.40	1	0.40 × 0.40	1
	C3	0.45 × 0.45	1	0.40 × 0.40	1	0.40 × 0.40	1	0.40 × 0.40	1
56–60	C1	0.45 × 0.45	1	0.40 × 0.40	1	0.40 × 0.40	1	0.40 × 0.40	1
	C2	0.45 × 0.45	1	0.40 × 0.40	1	0.40 × 0.40	1	0.40 × 0.40	1
	C3	0.45 × 0.45	1	0.40 × 0.40	1	0.40 × 0.40	1	0.40 × 0.40	1

Table notations: $\rho_{V}$ (vertical reinforcement ratio).

**Table 8:** Cost analysis.

	60 MPa	150 MPa	185 MPa	220 MPa
Concrete Volume (m^3^)	7882	6745	6494	7367
Steel Weight (Tons)	1217	1160	1102	1273
Concrete Cost, AED (USD)	2,286,004 (622,363)	3,710,047 (1,010,058)	3,896,766 (1,060,892)	4,789,144 (1,303,842)
Steel Cost, AED (USD)	3,529,441 (960,888)	3,364,513 (915,987)	3,197,987 (870,650)	3,693,968 (1,005,681)
Direct Cost, AED (USD)	5,815,445 (1,583,252)	7,074,560 (1,926,046)	7,094,753 (1,931,543)	8,483,112 (2,309,523)
Indirect Cost, AED (USD)	11,430,022 (3,111,818)	5,868,620 (1,597,729)	4,708,592 (1,281,912)	4,273,390 (1,163,428)
Total Cost, AED (USD)	17,245,467 (4,695,071)	12,943,180 (3,523,775)	11,803,345 (3,213,455)	12,756,502 (3,472,952)
